# Tetrahydroxy Stilbene Glucoside Promotes Mitophagy and Ameliorates Neuronal Injury after Cerebral Ischemia Reperfusion via Promoting USP10-Mediated YBX1 Stability

**DOI:** 10.1523/ENEURO.0269-24.2024

**Published:** 2024-10-24

**Authors:** Yuxian Li, Ke Hu, Jie Li, Xirong Yang, Xiuyu Wu, Qian Liu, Yuefu Chen, Yan Ding, Lingli Liu, Qiansheng Yang, Guangwei Wang

**Affiliations:** ^1^Medical College, Hunan University of Medicine, Huaihua, Hunan Province 418000, China; ^2^School of Basic Medical Sciences, Hunan University of Medicine, Huaihua, Hunan Province 418000, China; ^3^Department of Neurology, first affiliated hospital, Hunan University of Medicine, Huaihua, Hunan Province 418000, China; ^4^Biomedical Research Center, Hunan University of Medicine, Huaihua, Hunan Province 418000, China

**Keywords:** ischemic stroke, mitophagy, PINK1/Parkin, tetrahydroxy stilbene glucoside, USP10, YBX1

## Abstract

Tetrahydroxy stilbene glucoside (TSG) from *Polygonum multiflorum* exerts neuroprotective effects after ischemic stroke. We explored whether TSG improved ischemic stroke injury via PTEN-induced kinase 1 (PINK1)/Parkin-mediated mitophagy. Oxygen glucose deprivation/reoxygenation (OGD/R) in vitro model and middle cerebral artery occlusion (MCAO) rat model were established. Cerebral injury was assessed by neurological score, hematoxylin and eosin staining, 2,3,5-triphenyltetrazolium chloride staining, and brain water content. Apoptosis, cell viability, and mitochondrial membrane potential were assessed by flow cytometry, cell counting kit-8, and JC-1 staining, respectively. Colocalization of LC3-labeled autophagosomes with lysosome-associated membrane glycoprotein 2-labeled lysosomes or translocase of outer mitochondrial membrane 20-labeled mitochondria was observed with fluorescence microscopy. The ubiquitination level was determined using ubiquitination assay. The interaction between molecules was validated by coimmunoprecipitation and glutathione *S*-transferase pull-down. We found that TSG promoted mitophagy and improved cerebral ischemia/reperfusion damage in MCAO rats. In OGD/R-subjected neurons, TSG promoted mitophagy, repressed neuronal apoptosis, upregulated Y-box binding protein-1 (YBX1), and activated PINK1/Parkin signaling. TSG upregulated ubiquitin-specific peptidase 10 (USP10) to elevate YBX1 protein. Furthermore, USP10 inhibited ubiquitination-dependent YBX1 degradation. *USP10* overexpression activated PINK1/Parkin signaling and promoted mitophagy, which were reversed by *YBX1* knockdown. Moreover, TSG upregulated USP10 to promote mitophagy and inhibited neuronal apoptosis. Collectively, TSG facilitated PINK1/Parkin pathway-mediated mitophagy by upregulating USP10/YBX1 axis to ameliorate ischemic stroke.

## Significance Statement

Ischemic stroke is one of the leading causes of disability and death worldwide. Previous studies have demonstrated a neuroprotective role of tetrahydroxy stilbene glucoside (TSG) in ischemic stroke, while the underlying mechanism is still not fully understood. Here, this study confirmed that TSG relieved cerebral ischemia/reperfusion injury in vivo and in vitro via facilitated PINK1/Parkin-mediated mitophagy. In addition, we further identified the molecular mechanism by which TSG regulates mitochondrial autophagy. Our study provided new insights into the protective role of TSG in ischemic stroke via regulating mitophagy.

## Introduction

Ischemic stroke, induced by a sudden occlusion of the cerebral artery, is one of the leading causes of disability and death around the world. Recent therapeutic method includes rapid reperfusion using mechanical and pharmacological thrombolysis, both of which are time-sensitive. Furthermore, the recanalization of blood flow always elicits ischemia/reperfusion (I/R) damage (Tuo et al., 2022). Studies have confirmed that neuronal injury is an important pathogenesis of ischemic stroke (Zhao et al., 2022). Therefore, finding novel therapeutic medicines to rescue neuronal damage is significant for protecting the brain following stroke.

Mitophagy, as a selective autophagy, is a crucial biological process to remove redundant or dysfunctional mitochondria, thus maintaining energy metabolism (Shao et al., 2020). Mitophagy is a benefit for the function maintenance and survival of neurons, while excessive mitophagy can also cause cell death under certain circumstances; therefore, mitophagy plays double-edged roles in ischemic stroke (Song et al., 2022). PTEN-induced kinase 1 (PINK1)/Parkin signaling is an important mitophagy signaling in neurological diseases (J. Li et al., 2023a). PINK1 modulates the translocation of Parkin in damaged mitochondria and drives their elimination through mitophagy (Quinn et al., 2020). Activating mitophagy mediated by PINK1/Parkin signaling can improve neuronal damage caused by cerebral I/R (M. Wu et al., 2021, Mao et al., 2022). Thus, PINK1/Parkin-mediated mitophagy is an important therapeutic target against ischemic stroke.

Tetrahydroxy stilbene glucoside (TSG) is purified from medicinal herb *Polygonum multiflorum* and has been verified to exert powerful free radical scavenging, antioxidative and antiapoptotic activities (J. Wu et al., 2017). A current study has shown that TSG eliminated brain injury in a mice model of bilateral common carotid artery (CCA) occlusion (M. Li et al., 2022b). The neuroprotective effect of TSG has also been validated on a cell model of oxygen glucose deprivation/reoxygenation (OGD/R) and a rat I/R model of middle cerebral artery occlusion (MCAO; Ruan et al., 2021). TSG promoted neuronal mitophagy and inhibited apoptosis in ischemic stroke (Y. Li et al., 2021). Moreover, TSG promoted mitophagy via regulating PINK1/Parkin pathway to alleviate inflammatory damage (Gao et al., 2020). Nevertheless, the specific molecular mechanism through which TSG regulates mitophagy in ischemic stroke is still unclear.

Ubiquitin-specific peptidase 10 (USP10), one member of the ubiquitin-specific protease family, is a deubiquitination enzyme implicated in numerous essential cellular activities (Ye et al., 2022). Previous study has reported that USP10 suppressed apoptosis and inflammation to protect against cerebral ischemia damage (L. Wang et al., 2019b). USP10 alleviated neuroinflammation in ischemic stroke via repressing nuclear factor-κB signaling (C. Xie et al., 2023). Furthermore, USP10 was reported to participate in regulating autophagy in neurodegenerative diseases (Bhattacharya et al., 2020). Resveratrol, a TSG analog, has been reported to enhance USP10-mediated deubiquitination of p53 (Oi et al., 2015). Does TSG also play a role in promoting deubiquitination function of USP10?

Here, the molecular mechanism of TSG regulating mitophagy and neuronal injury following ischemic stroke was explored through the in vitro and in vivo experiments. We hypothesized that TSG upregulated USP10 to improve brain injury in ischemic stroke via promoting PINK1/Parkin-mediated mitophagy. Our study provided a better understanding of the molecular mechanism of TSG as a treatment for ischemic stroke.

## Materials and Methods

### PC12 cell culture and OGD/R

Rat PC12 cells were received from American Type Culture Collection. Cells were cultured in DMEM (Invitrogen) supplemented with 10% FBS, penicillin (100 U/mL), and streptomycin (100 U/mL), in a humidified incubator with 5% CO2 at 37°C.

PC12 cell subjected to OGD/R was constructed to mimic cerebral ischemic conditions. In brief, cells were maintained in a hypoxia chamber in glucose-free DMEM under 95% N2 and 5% CO_2_ for 2 h (Deng et al., 2022). Then cells were moved to normal culture conditions. After 24 h cultivation, cells were applied for the following assays.

For the treatment of TSG, cells were added with TSG (2, 4, or 8 μM, Yuanbaofeng Medical Technology) for 24 h incubation at the time of reperfusion. Cells were added with the proteasome inhibitor MG132 (25 μM, Sigma-Aldrich) or protein synthesis inhibitor cycloheximide (CHX; 50 μg/ml; Cell Signaling Technology, CST) and incubated for the indicated time periods.

### MCAO model construction and drug treatment

A total of 74 adult Sprague Dawley rats (male, 230–250 g, 6–8 weeks) were obtained from the Hunan University of Medicine. Animals were given water and food *ad libitum* and housed in a room with a 12 h light/dark cycle at 22°C. All animal procedures were performed in accordance with the National Institutes of Health Guidelines for Animal Research animal care committee's regulations and approved by our hospital.

To establish MCAO model, 54 animals were anesthetized with 5% isoflurane, followed by maintaining with 1.5% isoflurane. Warming pad was applied to maintain the body temperature at 37°C. After a ventral midline incision, the left CCA, external carotid artery (ECA) and internal carotid artery (ICA) were isolated. Then a monofilament nylon suture coated with silicone was gently inserted into the ICA through the ECA. After 2 h, the filament was withdrawn to allow reperfusion. Rats in the sham group received the same operation without artery occlusion. Next, the rat's neck was subcutaneously injected with TSG (10, 20, or 40 mg/kg) dissolved in saline at 4 h after reperfusion and then subcutaneously administered with TSG for 7 d, twice a day (Ruan et al., 2021). The same volume of saline was injected into the rats in model and sham groups. Nevertheless, four of the animals died during the experiment, which were excluded. After experiment, rats were killed, and brain tissues were immediately collected for subsequent detections. According to experimental requirements, samples were divided into four cohorts, which were used for neurological function/2,3,5-triphenyltetrazolium chloride (TTC) measurements, brain water content, pathological examination, and biochemical test, respectively.

### Cell transfection

Specific *shRNA targeting USP10* (*sh-USP10*) or *Y-box binding protein-1* (*YBX1*; *sh-YBX1*) was employed to knock down the *USP10* or *YBX1* gene, respectively. To overexpress *USP10*, we subcloned the pcDNA3.1 vector with the full length of *USP10* gene for establishing *pcDNA3.1-USP10*. The plasmids were obtained from GeneChem. Scramble *shRNA (sh-NC)* and *oe-NC* vectors were used as negative controls. Then, Lipofectamine 2000 (Invitrogen) was utilized to transfect these vectors into the cells.

### Neurological score

A five-point scale was used to quantify the neurological deficits as previously described (Ruan et al., 2021): 4, unable to move autonomously and unconsciousness, serious; 3, lean to the left, severe; 2, circling to the left, moderate; 1, unable to extend the left forepaw, slight; and 0, no observable deficit, normal. Two evaluators blinded to the experiment design were responsible for assessing the neurological function. For each rat, the score was calculated within 60 s and repeated three times. Then Kruskal–Wallis *H* test was used to analyze the scores (D. Yang et al., 2019).

### Cerebral infarct volume

Brain tissues were cut into coronal sections (2 mm thickness). Next, the sections were stained for 0.5 h at 37°C with 2% TTC solution (Thermo Fisher Scientific). Following immersed with paraformaldehyde (4%) overnight, the Image-Pro Plus software was applied to obtain the infarct sizes of the brain slices. Infarct volume (%) = [(contralateral hemisphere volume) − (ipsilateral undamaged volume)] × 100 / (contralateral hemisphere volume).

### Brain water content

Brain tissues were gathered from the rats after indicated treatments. Next, the dry weight approach was applied to determine the brain water content. In brief, brains collected from the rats were sectioned into coronal slices (5 mm thickness) and divided into the ischemic and nonischemic hemispheres. Then an analytical balance was employed to measure the wet weight of the ischemic hemisphere samples. Afterward, the brain tissue was maintained for 24 h at 105°C in an oven. Then an analytical balance was also used to determine the dry weight of the brain tissue (wet weight − dry weight) × 100 / wet weight = brain water content (%) (Hatashita et al., 1988).

### Hematoxylin and eosin staining

Brain tissues were incubated overnight at 4°C with paraformaldehyde (4%), before being sectioned into paraffin slices (5 μm). Later, following deparaffinized in xylene and rehydrated in gradient ethanol, slices were then stained by hematoxylin (2 g/l) for 5 min and then washed by distilled water. Slices were then incubated with eosin solution (1%) for 2 min, followed by washing with distilled water. Afterward, samples were sealed with neutral resin. Light microscopy (Olympus, Tokyo, Japan) was used to obtain the pictures of neuronal morphological alterations in the cerebral cortex.

### Immunohistochemistry

Brain tissues were placed in phosphate-buffered saline (PBS), followed by sectioning into frozen slices (5 µm). After incubation for 0.5 h in H_2_O_2_ (3%) for blocking peroxidase activity, slices were treated with 3% BSA at 37°C for 0.5 h. Next, they were immersed at 4°C in anti-LC3 (PA1-46286, 1:200, Invitrogen) overnight. Following incubation, slices were washed three times, prior to immersion for 1 h at 37°C in secondary antibody. Finally, after staining with diaminobenzidine and hematoxylin, sections were photographed by microscopy (BX51, Olympus).

### RT-qPCR

TRIzol reagent (Takara Bio) was used to extract the total RNA of brain tissues or cells. NanoDrop Spectrophotometer (BioDrop) was applied for measuring RNA concentration and purity. Subsequently, PrimeScript RT reagent kit (Takara Bio) was applied to operate reverse transcription reaction for obtaining cDNA. ABI 7300 Real-Time PCR System was used to perform quantitative RT-PCR with SYBR Green PCR premix (Thermo Fisher Scientific). The comparative threshold cycle method (2-DeltaDeltaCt) was used to calculate RNA expression levels normalized to GAPDH. The primers are displayed in Table 1.

**Table 1.** The primer sequences used in RT-qPCR

Gene	Forward primer (5′-3′)	Reversed primer (5′-3′)
*USP10*	*AGAGGAACTTCTGGACGGACAAG*	*CTCTGACCAAGACCACCAGC*
*YBX1*	*AAGTGATGGAGGGTGCTGAC*	*TGCCATCCTCTCTAGGCTGT*
*GAPDH*	*TGATGGGTGTGAACCACGAG*	*TCATGAGCCCTTCCACGATG*

### Western blot

Total proteins were extracted from brain tissues and cells with RIPA lysis buffer containing protease inhibitors and phosphatase inhibitors (Sigma-Aldrich). For the protein analysis of mitochondria, mitochondria were isolated from brain tissues (C3606, Beyotime) and cells (C3601, Beyotime) based on the kit instructions. The pellet containing separated mitochondria was resuspended in mitochondrial lysate with phenylmethanesulfonyl fluoride (1 mM). BCA assay (Solarbio) was used to determine protein concentrations. Next, equal amounts (30 μg) of protein samples were separated with SDS–PAGE, followed by movement to PVDF membranes. After blocking with skimmed milk (5%) for 1 h, membranes were immersed at 4°C overnight in primary antibodies against LC3II/I (PA1-46286, 1:1,000, Invitrogen), p62 (ab109012, 1:1,000, Abcam), PINK1 (PA1-16604, 1:500, Invitrogen), Parkin (702785, 1:200, Invitrogen), translocase of outer mitochondrial membrane 20 (Tomm20, ab186735, 1:1,000, Abcam), YBX1 (ab76149, 1:1,000, Abcam), GAPDH (ab181602, 1:10,000, Abcam), cytochrome C oxidase subunit IV (COX IV; ab202554, 1:2,000, Abcam), and USP10 (PA5-117656, 1:500, Invitrogen). Membranes were immersed for 1 h in secondary antibodies combined with horseradish peroxidase, followed by visualization with enhanced chemiluminescence reagent (Thermo Fisher Scientific). COX IV and GAPDH were used as internal references for mitochondrial and total fractions, respectively.

### Cell counting kit-8 (CCK-8) assay

The viability of PC12 cells was assessed using CCK-8 assay (C0037, Beyotime). Cells (2 × 10^3^ cells/100 μl) were planted to a 96-well plate, followed by the indicated treatments. Next, CCK-8 reagent (10 μl) was added into the cells for 2 h incubation at 37°C. Lastly, a microplate reader (Thermo Fisher Scientific) was used to detect the absorbances at 450 nm.

### Flow cytometry

Apoptosis in PC12 cells was detected by using Annexin V-FITC apoptosis detection kit (Beyotime). In brief, after indicated treatments, Annexin V-FITC (5 μl) and propidium iodide (10 μl) were added into the cells for 20 min incubation in darkness. Flow cytometer (ACEA Biosciences) was used to detect the apoptotic cells.

### Mitochondria membrane potential (MMP)

The alteration in MMP was detected by staining with JC-1 dye (Beyotime). In brief, cells were planted in 12-well plates. Following the corresponding treatment, 2 mmol/l JC-1 was added into the cells, before being incubated for 20 min with 5% CO2 at 37°C in an incubator. Next, after being rinsed in PBS, cells were observed under a fluorescence microscope, and the fluorescence images were obtained. An emission wavelength of 530 or 590 nm and an excitation wavelength of 490 or 525 nm were used for detecting JC-1 monomers or aggregates, respectively. A fluorescence spectrophotometer was applied to examine the fluorescent intensity. MMP was reflected by calculating the percentage of JC-1 aggregates (red) to monomer (green).

### Immunofluorescence

Brain sections were incubated at 4°C overnight with primary antibodies against NeuN (ab104224, Abcam) and YBX1 (ab76149, Abcam), following fixation, embedding, slicing, permeabilization, and blocking. Following incubation at 37°C for 1 h with secondary antibodies, sections were stained by DAPI. Fluorescence pictures were obtained utilizing fluorescence microscope (Olympus). PC12 cells in vitro were coincubated in primary antibodies against lysosome-associated membrane glycoprotein 2 (LAMP2, L0668, Sigma-Aldrich) and LC3 (83506, CST) or Tomm20 (ab186735, Abcam) and LC3, and other procedures were the same as above**.**

### Ubiquitination assay

HA-Ub plasmid (2 μg) was transfected into cells. After 48 h, cells were pretreated for 6 h with 30 μM of MG132, followed by resuspension in IP lysis buffer. Samples were incubated for 5 min at 4°C before being sonicated on ice. Following centrifugation for 15 min at 12,000 rpm, supernatant was incubated overnight at 4°C with anti-YBX1 (ab76149, Abcam) and then immersed at 4°C with protein A/G-Sepharose beads for another 2 h. Immunoprecipitated protein complexes on beads were subsequently eluted for 10 min at 95°C. Next, they were examined using Western blot with anti-YBX1 and anti-HA (H6908, Sigma-Aldrich).

### Coimmunoprecipitation

Cell lysates from PC12 cells were centrifugated at 4°C for 10 min at 13,000 × *g*. Next, after precleared for 2 h in protein A/G agarose beads (20 μl), the supernatants were immersed at 4°C in anti-USP10 (PA5-117656, 1:200, Invitrogen) or IgG (ab172730, 1:100, Abcam) overnight, prior to incubation for 2 h at 4°C in protein A/G plus agarose. The immunoprecipitated complexes were eluted through boiling with SDS loading buffer, after being washed in IP buffer. Next, primary antibodies including anti-USP10 and anti-YBX1 were used for the immunoblot analyses.

### Glutathione *S*-transferase (GST) pull-down

Recombinant glutathione transferase-USP10 (GST-USP10) was prepared in *E. coli* for GST pull-down assay. GST proteins were purified using glutathione Sepharose 4B microspheres (Solarbio) and then incubated with PC12 cells for 2 h. Then GST-USP10 fusion proteins or GST (Proteintech) on microspheres were added to the total lysate of PC12 cells and incubated overnight at 4°C on a rolling shaker. The bound proteins were eluted from the beads. Anti-USP10 and anti-YBX1 were used to carry out Western blot analysis.

### Statistical analysis

GraphPad Prism 7.0 was applied for performing statistical analyses. Each experiment was repeated at least three times. All data were exhibited as mean ± SD. One-way ANOVA followed by the Tukey's post hoc test was used to carry out the comparison of multiple groups, while Student's *t* test was used to operate the comparison between two groups. *p *< 0.05 was considered as significance.

## Results

### TSG improved cerebral I/R injury and promoted mitophagy in MCAO rats

**Figure 1.**
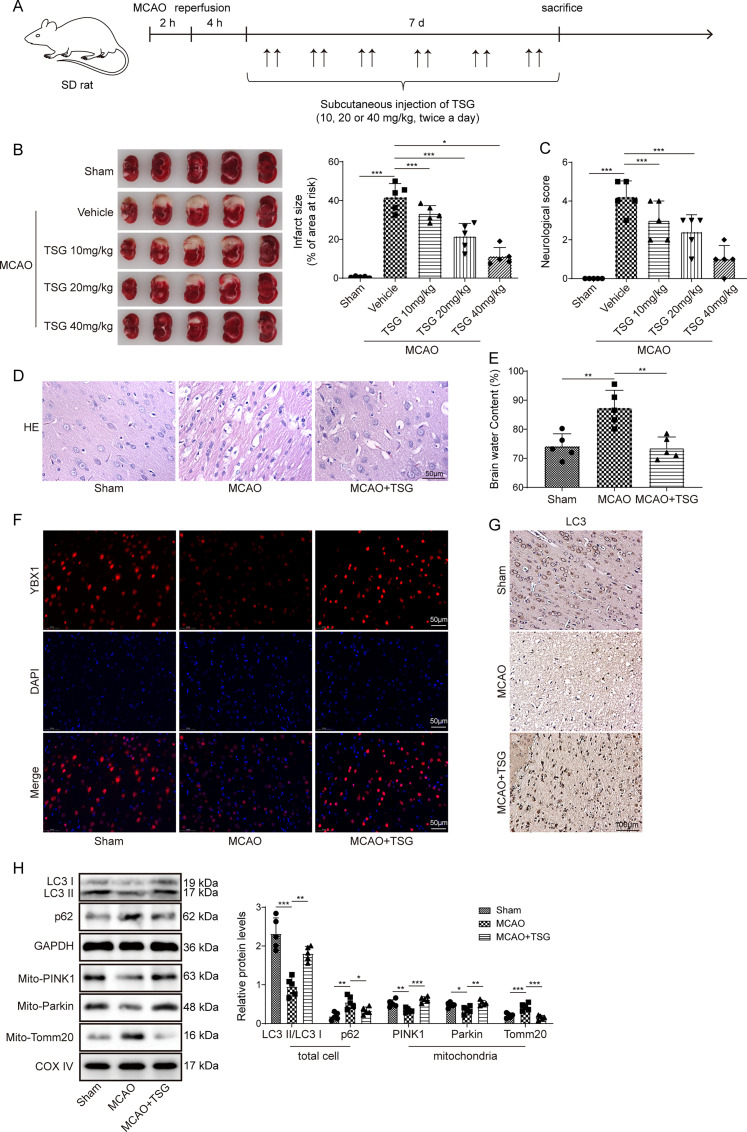
TSG improved cerebral I/R damage and promoted mitophagy in MCAO rats. ***A***, Schematic diagram of the experimental design. ***B***,***C***, Rats were used to construct MCAO model, followed by subcutaneous injection of TSG (10, 20, or 40 mg/kg). ***B***, TTC staining for determining infarction volume. ***C***, Neurological scores. ***D–H***, Rats were subjected to MCAO, followed by treatment with TSG (40 mg/kg). ***D***, Hematoxylin and eosin staining for observing the morphological changes of cortical cells. ***E***, Brain edema determined with the ratio of dry/wet weight. ***F***, Immunofluorescence for detecting the YBX1 expression. ***G***, Immunohistochemistry for detection of LC3 expression. ***H***, Western bolt for detecting mitophagy-associated proteins (LC3II/I, p62, PINK1, Parkin) and Tomm20 (a mitochondrial marker). *n* = 5 per group. *p < 0.05; **p < 0.01; ***p < 0.001. Data are displayed as mean ± SD.

First, we explored the neuroprotective effect of TSG on ischemic stroke. Male SD rats were used to construct MCAO model. Following 2 h of occlusion, the filament was withdrawn for reperfusion. Rats were then treated with TSG (10, 20, or 40 mg/kg) dissolved in saline via subcutaneous injection at 4 h after reperfusion and then for another 6 d (twice a day). Besides, rats in model and sham groups were administrated with the same volume of saline. A timeline image of the experimental design is showed in Figure 1*A*. Compared with the sham group, MCAO rats exhibited significant cerebral infarction, while treatment with TSG (10, 20, or 40 mg/kg) decreased the infarction volume (Fig. 1*B*). Besides, in a dose-dependent manner, TSG treatment significantly reduced the neurological scores after MCAO (Fig. 1*C*), indicating that TSG alleviated MCAO-induced neurological deficit. According to the above data, 40 mg/kg of TSG had the strongest effects, which was selected for subsequent experiments. Hematoxylin and eosin staining showed that MCAO caused neuronal vacuolation in the infarct area, whereas TSG ameliorated the damage of cortical neurons (Fig. 1*D*). MCAO caused significant brain edema, while TSG treatment reduced brain water content in MCAO rats (Fig. 1*E*). Immunofluorescence showed that MCAO caused decreases in the immunofluorescence intensity of YBX1 in the cerebral cortex, which were increased after TSG treatment (Fig. 1*F*). We then explored whether mitophagy was implicated in the protective impact of TSG. Immunohistochemistry showed that MCAO downregulated the expression of LC3 (an autophagy marker) in brain tissues, while TSG treatment upregulated LC3 expression (Fig. 1*G*). It was found that MCAO downregulated LC3 and upregulated p62 protein levels in brain tissues, which was reversed by TSG treatment (Fig. 1*H*). It is known that PINK1/Parkin signaling is activated during mitophagy, and PINK1 recruits Parkin to the mitochondria surface to activate Parkin E3 ubiquitin ligase (Nguyen et al., 2016). Parkin can label mitochondrial components such as Tomm20 through ubiquitination, which results in the degradation of these mitochondrial proteins by proteasome (Stolz et al., 2014). To further verify the effect of TSG on mitophagy, we isolated the mitochondria, and the results displayed that MCAO decreased mitochondrial Parkin and PINK1 protein levels but upregulated mitochondrial Tomm20 in brain tissues, while TSG abolished the effects caused by MCAO (Fig. 1*H*). These results indicated that TSG ameliorated MCAO-induced cerebral ischemic injury and facilitated mitophagy.

### TSG promoted mitophagy and inhibited apoptosis by upregulating YBX1 and activating PINK1/Parkin signaling in OGD/R-induced neurons

**Figure 2.**
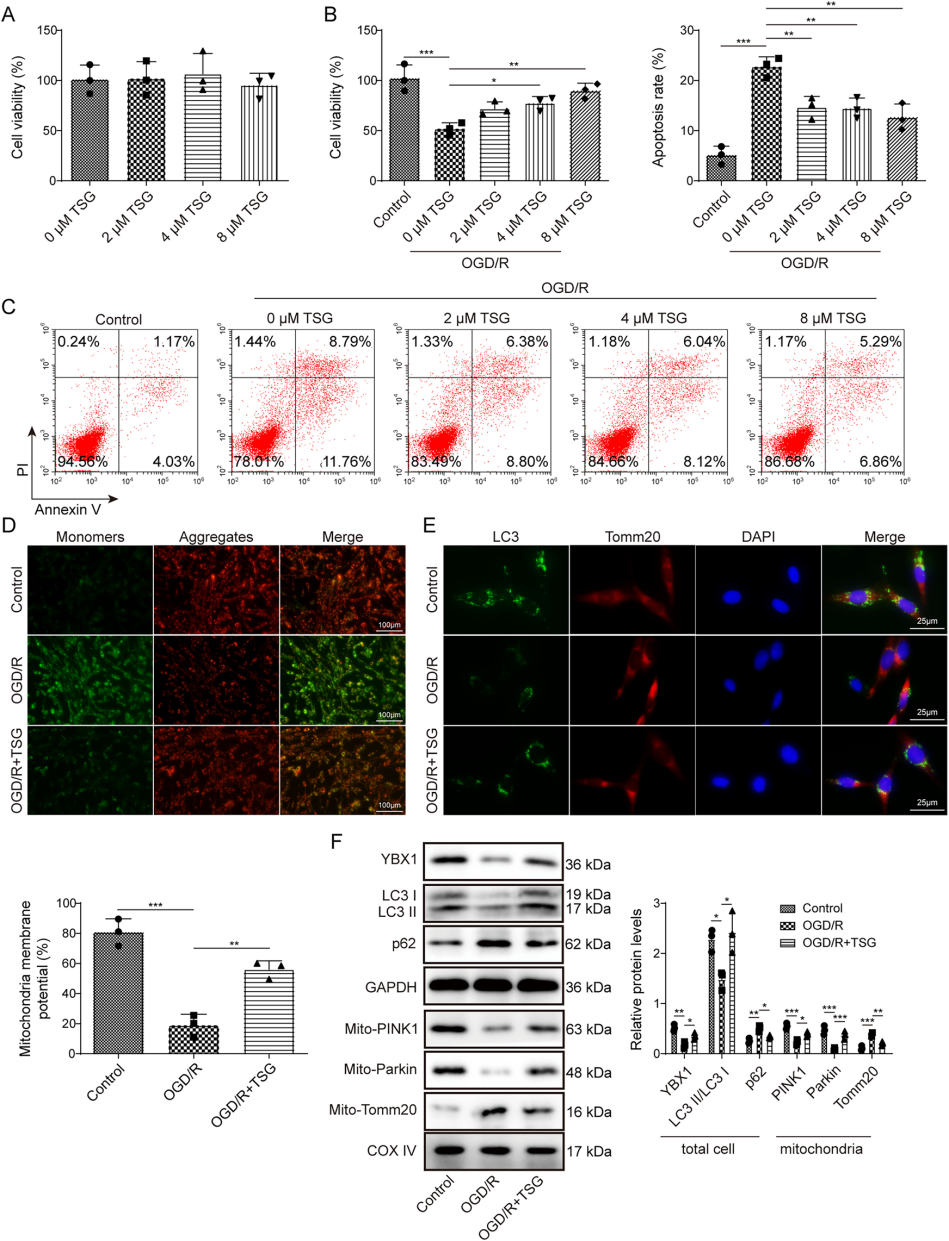
TSG promoted mitophagy and inhibited apoptosis by upregulating YBX1 and activating PINK1/Parkin signaling in OGD/R-induced neurons. ***A***, Cell viability was determined with CCK-8 in PC12 cells treated with TSG (0, 2, 4, or 8 μM). ***B***, ***C***, PC12 cells were subjected to OGD/R, followed by the treatment of TSG (2, 4, or 8 μM). ***B***, CCK-8 for measuring cell viability. ***C***, Apoptosis detected using flow cytometry. ***D–F***, PC12 cells were exposed to OGD/R, prior to TSG (8 μM) incubation. ***D***, JC-1 staining for detecting MMP. ***E***, Immunofluorescence for measuring the colocalization of LC3 and Tomm20. ***F***, Western blot for detecting autophagy-associated proteins (LC3II/I, p62, PINK1, Parkin), Tomm20 (a mitochondrial marker), and YBX1. *n* = 3. *p < 0.05; **p < 0.01; ***p < 0.001. Data are displayed as mean ± SD.

We then investigated the potential mechanism underlying the neuroprotection of TSG. PC12 cells were treated with TSG (0, 2, 4, 8 μM). The data showed that no obvious alteration was observed in cell viability following the treatments of different doses of TSG, suggesting that TSG had no toxic impact on PC12 cells in the range of 0–8 μM (Fig. 2*A*). Then PC12 cells were exposed to OGD/R, followed by the treatment of TSG (2, 4, or 8 μM). Compared with the control group, cell viability was decreased by OGD/R, while TSG treatment elevated cell viability in a dose-dependent manner (Fig. 2*B*). OGD/R promoted apoptosis of PC12 cells, while TSG repressed apoptosis in a dose-dependent manner (Fig. 2*C*). Mitophagy-mediated removal of injured mitochondria is critical for cell homeostasis and mitochondrial function (Tian et al., 2022). Here, we examined mitochondrial function by assessing MMP using JC-1 staining. PC12 cells were exposed to OGD/R, prior to the addition of TSG (8 μM). Compared with the control group, OGD/R decreased MMP, whereas TSG treatment increased MMP (Fig. 2*D*). Moreover, OGD/R decreased the colocalization of Tomm20 and LC3 in PC12 cells, which was increased after the treatment of TSG (Fig. 2*E*). PINK1 activation induced by mitochondrial injury motivates phosphorylation-dependent Parkin activation and ubiquitin-dependent mitochondrial removal through mitophagy (Agarwal and Muqit, 2022). Next, whether PINK1/Parkin pathway participated in TSG-caused mitophagy was explored through measuring the expressions of mitophagy-related proteins. Compared with the control group, OGD/R decreased LC3II/I and YBX1, increased p62, downregulated mitochondrial PINK1 and Parkin, and increased mitochondrial Tomm20 in PC12 cells; however, TSG treatment overturned the protein expressions in OGD/R-subjected PC12 cells (Fig. 2*F*). These outcomes demonstrated that TSG could facilitated mitophagy and suppressed apoptosis in OGD/R-induced neurons through upregulating YBX1 and activating PINK1/Parkin signaling.

### TSG increased YBX1 protein expression in OGD/R-subjected neurons via upregulating USP10

**Figure 3.**
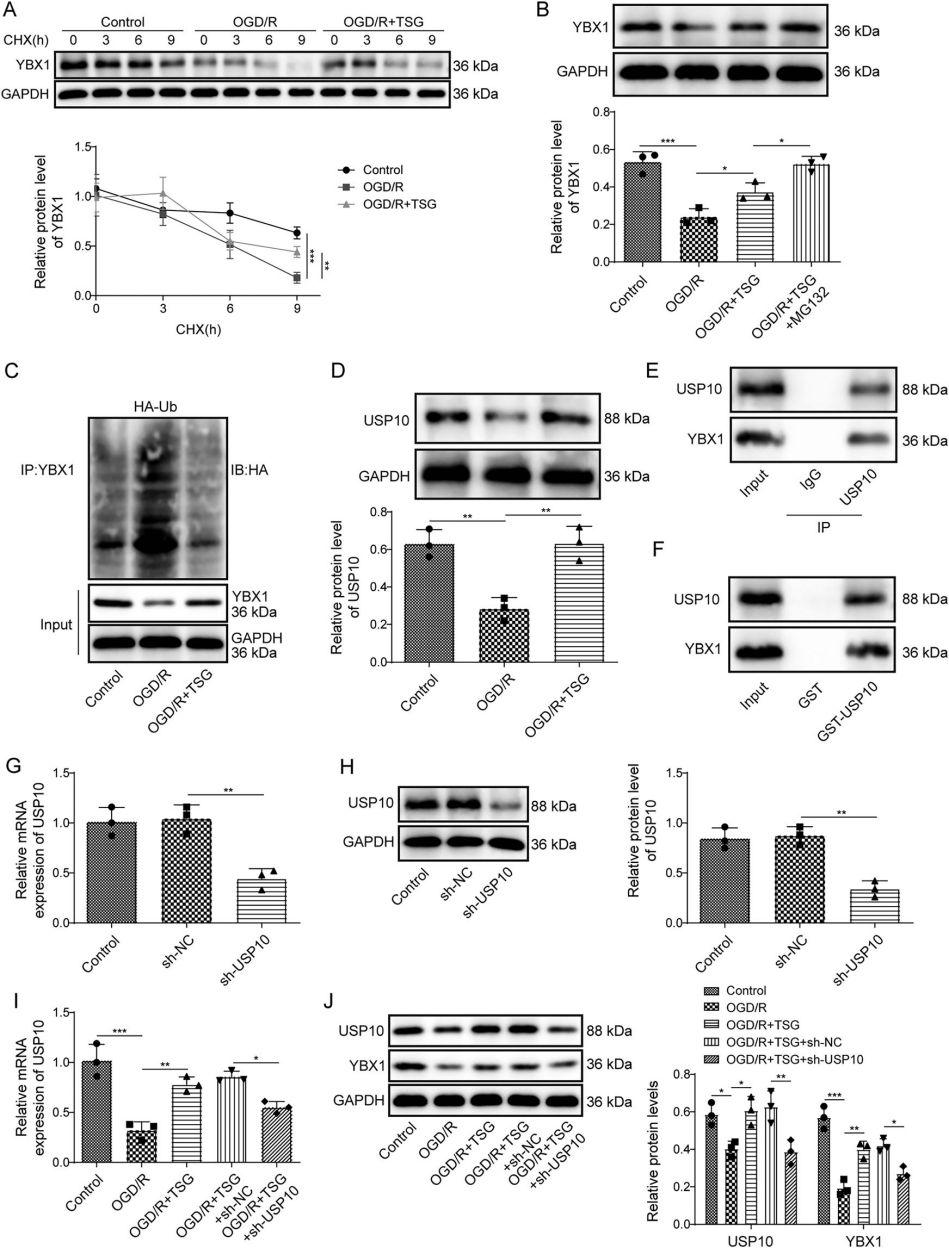
TSG increased YBX1 protein expression in OGD/R-subjected neurons via upregulating USP10. ***A–D***, PC12 cells were exposed to OGD/R, prior to TSG (8 μM) incubation. ***A***, Western blot for determining the YBX1 protein level after the treatment of CHX. ***B***, Western blot for testing the YBX1 protein level after the treatment of MG132. ***C***, Detection of the YBX1 ubiquitination level. ***D***, Western blot for detecting the USP10 protein level. ***E***, ***F***, Coimmunoprecipitation (***E***) and GST pull-down (***F***) for determining the interaction of USP10 and YBX1. ***G***, ***H***, *sh-USP10* was transfected into PC12 cells. RT-qPCR and Western blot for detecting USP10 expressions. ***I***, ***J***, PC12 cells were transfected by *sh-USP10*, before being exposed to OGD/R or treated with TSG. ***I***, RT-qPCR for determining *USP10* mRNA expression. ***J***, Western blot for testing USP10 and YBX1 protein levels. *n* = 3. *p < 0.05; **p < 0.01; ***p < 0.001. Data are displayed as mean ± SD.

Next, we examined the possible mechanisms by which TSG upregulates YBX1. YBX1 protein levels in PC12 cells after the treatment of protein synthesis inhibitor (CHX) were detected using Western blot. Compared with the control group, YBX1 protein degradation was rapid in OGD/R-exposed neurons, while TSG treatment slowed down the degradation of YBX1 protein (Fig. 3*A*). Furthermore, YBX1 protein levels in PC12 cells after the treatment of proteasome inhibitor (MG132) were detected using Western blot. The YBX1 protein level was downregulated in OGD/R-exposed cells, while it was significantly upregulated after TSG treatment; however, MG132 treatment further elevated the YBX1 protein level (Fig. 3*B*). The above results indicated that TSG could stabilize OGD/R-induced degradation of YBX1 protein. Moreover, ubiquitination assay showed that OGD/R promoted the ubiquitination of YBX1 in PC12 cells, which was inhibited after TSG treatment (Fig. 3*C*). Compared with the control group, the USP10 protein level was decreased in OGD/R-exposed cells, whereas TSG treatment reversed OGD/R-induced decrease of USP10 (Fig. 3*D*). In PC12 cells, the direct interaction between USP10 and YBX1 was validated using coimmunoprecipitation (Fig. 3*E*). Moreover, GST pull-down further confirmed the relationship of USP10 and YBX1 (Fig. 3*F*). Subsequently, PC12 cells were transfected with *sh-USP10*, and the silencing efficiency was validated, as indicated by the data that *sh-USP10* transfection downregulated USP10 at mRNA and protein levels (Fig. 3*G*,*H*). Then PC12 cells that transfected with *sh-USP10* were subjected to OGD/R and treated with TSG. In OGD/R-exposed cells, TSG treatment upregulated USP10 at mRNA and protein levels; however, *USP10* downregulation reversed this effect induced by TSG (Fig. 3*I*,*J*). Moreover, in OGD/R-subjected cells, knockdown of *USP10* reversed the upregulated the YBX1 protein level caused by TSG (Fig. 3*J*). Collectively, TSG inhibited YBX1 ubiquitination degradation by upregulating USP10 in OGD/R-exposed neurons.

### *USP10* overexpression inhibited ubiquitination-dependent degradation of YBX1 in OGD/R-subjected neurons

**Figure 4.**
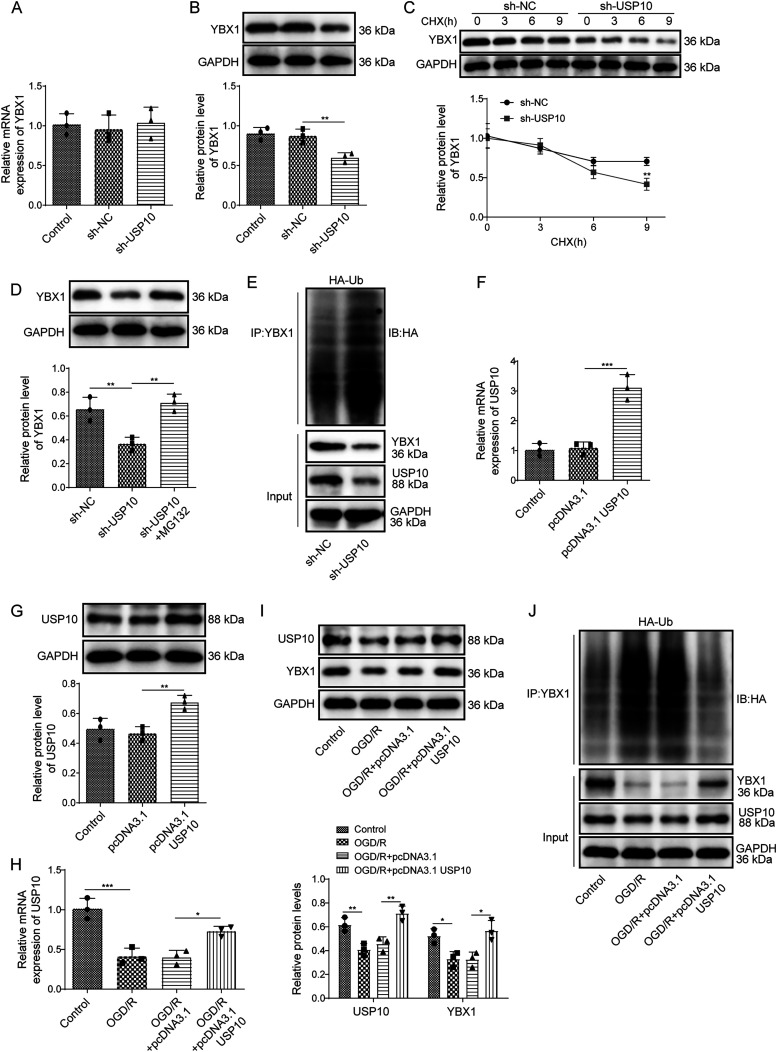
*USP10* overexpression inhibited ubiquitination-dependent degradation of YBX1 in OGD/R-subjected neurons. ***A–E***, *sh-USP10* was transfected into PC12 cells. ***A***, RT-qPCR for determining *YBX1* mRNA expression. ***B***, Western blot for detecting the YBX1 protein level. ***C***, Western blot for testing the YBX1 protein level after treatment with CHX. ***D***, Western blot for determining the YBX1 protein level after MG132 treatment. ***E***, The ubiquitination level of YBX1. ***F***, ***G***, *pcDNA 3.1-USP10* was transfected into PC12 cells. RT-qPCR and Western blot were applied for measuring USP10 levels. ***H–J***, *pcDNA-3.1-USP10* was transfected into PC12 cells, followed by OGD/R exposure. ***H***, RT-qPCR for detecting the *USP10* mRNA level. ***I***, Western blot for determining USP10 and YBX1 protein levels. ***J***, The ubiquitination level of YBX1. *n* = 3. *p < 0.05; **p < 0.01; ***p < 0.001. Data are displayed as mean ± SD.

Our study further examined how USP10 regulated YBX1 expression in PC12 cells. Knockdown of *USP10* in PC12 cells did not affect the *YBX1* mRNA level (Fig. 4*A*); however, *USP10* silencing downregulated the protein level of YBX1 (Fig. 4*B*). With the treatment of CHX, knockdown of *USP10* accelerated the degradation of YBX1 protein (Fig. 4*C*). Furthermore, compared with the *sh-NC* group, knockdown of *USP10* reduced the YBX1 protein level, while YBX1 protein degradation induced by *USP10* knockdown was repressed in the presence of MG132 (Fig. 4*D*). Meanwhile, ubiquitination assay exhibited that knockdown of USP10 promoted the ubiquitination of YBX1 in neurons (Fig. 4*E*). Subsequently, cells were transfected with *pcDNA 3.1-USP10*, and the transfection efficiency was validated. As shown in Figure 4, *F* and *G*, *pcDNA 3.1-USP10* upregulated *USP10* mRNA and protein levels (Fig. 4*F*,*G*). Then PC12 cells were transfected with *pcDNA 3.1-USP10*, followed by OGD/R exposure. OGD/R downregulated USP10 at mRNA and protein levels, while overexpression of *USP10* upregulated USP10 (Fig. 4*H*,*I*); besides, OGD/R declined the protein level of YBX1, whereas *USP10* overexpression increased the YBX1 protein level (Fig. 4*I*). Moreover, OGD/R promoted the ubiquitination of YBX1, which was reversed after overexpression of *USP10* (Fig. 4*J*). The above indicated that in OGD/R-exposed neurons, USP10 suppressed YBX1 protein ubiquitination degradation.

### Knockdown of *YBX1* reversed the effects of *USP10* overexpression on PINK1/Parkin signaling and mitophagy in OGD/R-exposed neurons

**Figure 5.**
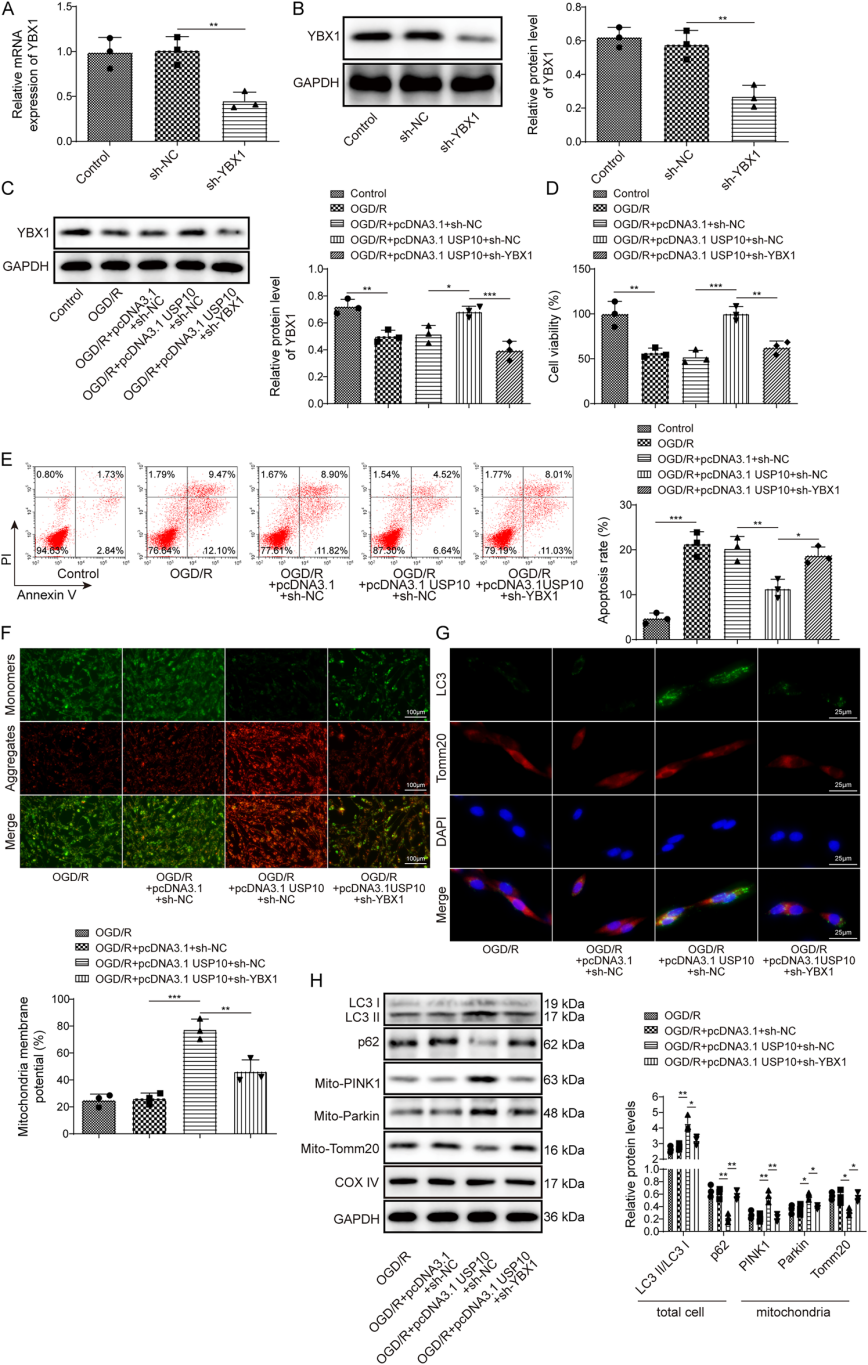
Knockdown of *YBX1* reversed the effects of USP10 overexpression on PINK1/Parkin signaling and mitophagy in OGD/R-exposed neurons. ***A–B***, *sh-YBX1* was transfected into PC12 cells. RT-qPCR and Western bolt for detecting YBX1 expression. ***C–H***, PC12 cells were transfected with *sh-NC*, *sh-YBX1*, *pcDNA- 3.1*, or *pcDNA-3.1-USP10*, followed by exposure to OGD/R. ***C***, Western blot for detection of the YBX1 protein level. ***D***, CCK-8 assay for cell viability measurement. ***E***, Flow cytometry for determining apoptosis. ***F***, JC-1 staining for measuring MMP. ***G***, Immunofluorescence for detecting the colocalization of LC3 and Tomm20. ***H***, Western blot for determining autophagy-associated proteins (LC3II/I, p62, PINK1, Parkin) and Tomm20 (a mitochondria marker). *n* = 3. *p < 0.05; **p < 0.01; ***p < 0.001. Data are displayed as mean ± SD.

Then, we investigated whether USP10 affected PINK1/Parkin signaling and neuronal mitophagy via YBX1. *sh-YBX1* transfection in PC12 cells reduced YBX1 mRNA and protein levels (Fig. 5*A*,*B*), indicating successful knockdown of YBX1. Subsequently, PC12 cells were transfected with *sh-YBX1* or *pcDNA-3.1-USP10*, followed by exposure to OGD/R. The YBX1 protein level was decreased in OGD/R-subjected neurons, which was upregulated by USP10 overexpression; however, the effect induced by USP10 overexpression was reversed after knockdown of *YBX1* (Fig. 5*C*). The decreased cell viability induced by OGD/R was elevated after overexpression of *USP10*, whereas *YBX1* silencing abolished the effect of *USP10* upregulation (Fig. 5*D*). The promoted cell apoptosis caused by OGD/R was repressed by *USP10* overexpression, which was reversed after knockdown of *YBX1* (Fig. 5*E*). Moreover, overexpression of *USP10* elevated MMP under OGD/R conditions, which was offset by *YBX1* silencing (Fig. 5*F*). Immunofluorescence showed that *USP10* overexpression enhanced the colocalization of LC3 and Tomm20, whereas this effect was abolished by overexpression of *USP10* (Fig. 5*G*). In addition, upregulation of *USP10* elevated LC3II/I and mitochondrial PINK1 and Parkin but downregulated the protein levels of p62 and mitochondrial Tomm20 under OGD/R conditions, whereas knockdown of *YBX1* reversed the effects of USP10 overexpression (Fig. 5*H*). The results suggested that USP10 activated PINK1/Parkin signaling and promoted neuronal mitophagy via upregulating YBX1 in OGD/R-exposed neurons.

### TSG heightened mitophagy and inhibited neuronal apoptosis through upregulating USP10 in OGD/R-induced neurons

**Figure 6.**
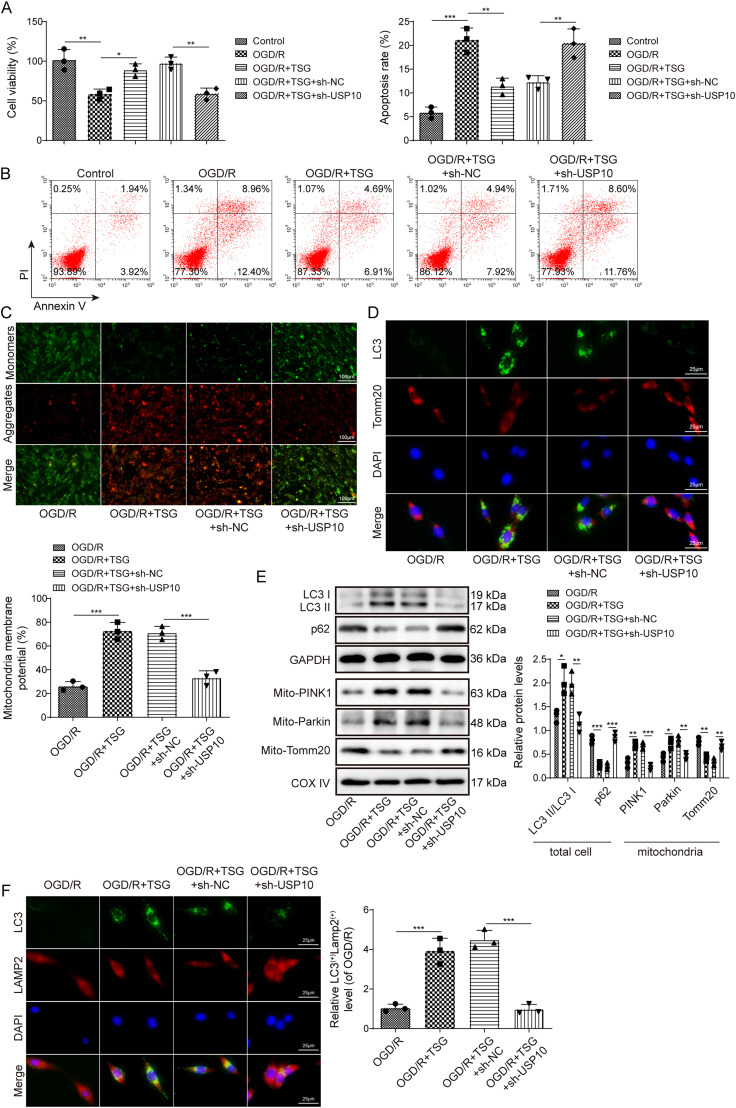
TSG heightened mitophagy and inhibited neuronal apoptosis through upregulating USP10 in OGD/R-induced neurons. *sh-NC* or *sh-USP10* was transfected into PC12 cells, before being exposed to OGD/R and treated with TSG. ***A***, CCK-8 assay for cell viability detection. ***B***, Apoptosis measured with flow cytometry. ***C***, JC-1 staining for determining MMP. ***D***, Immunofluorescence for detecting the colocalization of LC3 and Tomm20. ***E***, Western blot for testing autophagy-associated proteins (LC3II/I, p62, PINK1, Parkin) and Tomm20 (a mitochondria marker). ***F***, Immunofluorescence for detecting the colocalization of LAMP2 and LC3. *n* = 3. Data are shown as mean ± SD. *p < 0.05; **p < 0.01; ***p < 0.001.

To study whether USP10 was implicated in the neuroprotection of TSG on regulating mitophagy and neuronal apoptosis, cells were transfected with *sh-USP10*, followed by exposure to OGD/R and treated with TSG. We found that knockdown of *USP10* reversed the promoting effect of TSG on OGD/R-induced PC12 cell viability (Fig. 6*A*). TSG treatment rescued apoptosis in OGD/R-induced PC12 cells, whereas *USP10* knockdown reversed the effect induced by TSG (Fig. 6*B*). Furthermore, TSG treatment elevated MMP in OGD/R-exposed cells, which was abolished after knockdown of *USP10* (Fig. 6*C*). The enhanced colocalization of LC3 and Tomm20 caused by TSG treatment in OGD/R-exposed cells was reversed by *USP10* knockdown (Fig. 6*D*). As expected, knockdown of *USP10* abolished the upregulation of LC3II/I and mitochondrial PINK1 and Parkin, and downregulation of p62 and mitochondrial Tomm20 caused by TSG in OGD/R-subjected cells (Fig. 6*E*). LAMP2 is one of the major protein components of the lysosomal membrane (Qiao et al., 2023). The colocalization of LC3B-II with LAMP2 indicates fusion of autophagosome with lysosomes (H. Yang et al., 2020). Herein, we found that TSG increased the colocalization puncta of LC3 and LAMP2 in OGD/R-exposed cells, which was reversed after knockdown of *USP10* (Fig. 6*F*). Taken together, TSG upregulated USP10 to promote mitophagy and inhibit neuronal apoptosis in OGD/R-induced neurons.

## Discussion

Ischemic stroke is a major cause of neurological disability and mortality worldwide (Ahsan et al., 2021). Given that mitophagy is linked with many central nervous system disorders including cerebral ischemic injury (Guan et al., 2018), this research aims to investigate whether TSG plays a neuroprotective role in cerebral ischemic damage via regulating mitophagy and explores the potential mechanism. Here, we uncovered that TSG improved cerebral I/R injury and promoted mitophagy in MCAO rats. Meanwhile, TSG inhibited neuronal apoptosis and promoted mitophagy in OGD/R-induced neurons. Mechanically, TSG upregulated USP10 to elevate YBX1 protein expression, thereby facilitating PINK1/Parkin-mediated mitophagy and improving ischemic stroke injury.

Mitophagy controls the number and the quality of mitochondria, clearing excessive or dysfunctional mitochondria which may result in cell death (L. Li et al., 2023b). In the context of autophagy, the cytosolic LC3 form (nonlipidated LC3I) becomes linked to phosphatidylethanolamine, resulting in the lipidated LC3 (LC3II) form, which is aggregated on the autophagosomal membrane as an autophagy marker (M. Li et al., 2016). The colocalized dots of LC3 with LAMP2 (a marker of lysosome) are known as autolysosomes, reflecting the quantity of cargo to be degraded by autophagy (Liao et al., 2022). The overlap of LC3-labeled autophagosomes with Tomm20-labeled mitochondria indicates the level of mitophagy (X. Yang et al., 2018). Previous studies have indicated reperfusion following cerebral ischemia is an essential turning point for autophagy/mitophagy from the protecting mechanism to the detrimental process (Feng et al., 2017, 2018a). Moreover, existing evidence suggests the dual roles of mitophagy in ischemic stroke (Guan et al., 2018). During cerebral ischemic injury, mitophagy can be enhanced (L. Yang et al., 2022, Zhou et al., 2023) or blocked (Feng et al., 2018b, L. Li et al., 2023b) following the treatments of different drugs, while these drugs may exert neuroprotective functions; however, the precise mechanism remains unclarified. Here, whether TSG improved ischemic stroke injury through mitophagy was explored. Notably, concurrently with the neuroprotection of TSG, LC3II/I was increased, and the expression of p62 was decreased. Besides, the colocalized dots of LC3 with Tomm20 (a mitochondrial marker) in the cytoplasm were elevated after TSG treatment. Our research also found that MMP was increased with TSG treatment under OGD/R in vitro. These discoveries indicated that the enhanced mitophagy induced by TSG might be a crucial mechanism underlying the protective function.

PINK1/Parkin-mediated mitophagy has been reported to play a critical role in the neuroprotection following ischemic stroke (H. Wang et al., 2019a, Mao et al., 2022). When mitophagy occurs, PINK1/Parkin signaling is activated, and PINK1 recruits Parkin to the mitochondria surface to activate Parkin E3 ubiquitin ligase; Parkin can label mitochondrial components such as Tomm20 through ubiquitination, which results in the degradation of these mitochondrial proteins by proteasome (Stolz et al., 2014). For example, Lixuan Yang et al. have discovered that mitophagy was repressed in LPS-induced astrocytes, and Parkin in the cytoplasm failed to transfer to the mitochondria, which contributed to the stability of Tomm20; *Morinda officinalis* leads to ubiquitination and degradation of Tomm20 through promoting the enrichment of Parkin in mitochondria, thereby facilitating mitophagy (L. Yang et al., 2023). In addition, it has been revealed that BDNF induced mitophagy via reducing Tomm20 and increasing LC3II/LC3I, thereby alleviating high-glucose–induced brain microvascular endothelial cell injury (Jin et al., 2019). Our study discovered reduction of Parkin and increase of Tomm20 in mitochondria under OGD/R conditions. However, TSG could promote the transfer of Parkin to mitochondria, which led to the downregulation of Tomm20 in mitochondria, indicating activation of PINK1/Parkin-mediated mitophagy might be implicated in the neuroprotection of TSG on ischemic stroke.

Ubiquitination is usually mediated by ubiquitin proteasome system, while ubiquitin, ubiquitin protein ligase, ubiquitin-conjugating enzyme, ubiquitin-activating enzyme, deubiquitinase, and the proteasome are essential components of the ubiquitin proteasome system (Kane et al., 2021). USP10, a critical member of the deubiquitinase family, has been verified to be involved in many cellular processes and multiple neurodegenerative disorders (Bhattacharya et al., 2020). USP10 modulates pivotal signals involved in cell proliferation, apoptosis, and autophagy (H. Li et al., 2022a). Increase in USP10 level induced by vagus nerve stimulation was associated with the deubiquitination of IkappaB kinase (IKK)-γ in ischemic stroke (C. Xie et al., 2023). Encouraging USP10-dependent Smad4 deubiquitination aggravated cardiac fibrosis post myocardial I/R damage (S. Xie et al., 2022). Herein, we uncovered that the promoted mitophagy and inhibited neuronal apoptosis induced by TSG treatment could be reversed following knockdown of USP10 in OGD/R-exposed neurons. USP10 may be a molecular target for the neuroprotection of TSG, and this hypothesis can be further validated at the animal level in the future. Nevertheless, how TSG leads to USP10 activation is still unclear. It has been found that resveratrol, a TSG analog, disrupted the interaction between Ras-GTPase–activating protein SH3 domain-binding protein 1 (G3BP1) and USP10 through directly targeting G3BP1, thereby enhancing USP10-mediated p53 deubiquitination (Oi et al., 2015). G3BP1 is one of the main components of stress granules (SGs; Q. Wang et al., 2024). SGs are cytoplasmic aggregates of stalled translational preinitiation complexes induced by various environmental stresses such as heat shock, hypoxia, endoplasmic reticulum stress, and oxidative stress (An et al., 2019). In I/R injury, regulating the formation of SGs is a promising intervention for improving self-protection capability of the cells and inhibiting apoptosis (Si et al., 2020). As a resveratrol analog, whether TSG affects the activation of USP10 through directly interacting with G3BP1 protein or regulates the function of USP10 via combining with other proteins remains to be further explored.

YBX1, a DNA/RNA-binding protein, is one member of the cold-shock protein family (Mordovkina et al., 2020). It has been demonstrated that upregulating YBX1 protein with dexmedetomidine could protect neuronal cells from I/R-induced apoptosis (Yan et al., 2023); YBX1 protected against apoptosis induced by OGD/R in PC12 cells (Tuerxun et al., 2021). Besides, YBX1 carried by neural stem cell extracellular vesicles inhibited neuronal pyroptosis in ischemic stroke (Peng et al., 2023). Furthermore, *YBX1* knockdown decreased PINK1 and inhibited mitophagy during embryonic development (Jiang et al., 2023). Additionally, YBX1 enhanced PINK1/Parkin-mediated mitophagy, thereby promoting brown adipogenesis (R. Wu et al., 2022). Here, we found that *USP10* overexpression inhibited ubiquitination-dependent degradation of YBX1 in OGD/R-exposed neurons. Furthermore, *USP10* overexpression promoted PINK1/Parkin signaling activation and neuronal mitophagy; however, these effects could be reversed by knockdown of *YBX1*, indicating that USP10 upregulated YBX1 to activate PINK1/Parkin signaling and promote neuronal mitophagy.

To sum up, TSG played a vital protective role in cerebral ischemic damage via upregulating USP10/YBX1 axis. USP10 may be a molecular target for the neuroprotection of TSG. Inevitably, there are also some limitations. First, no further intervention studies have been designed to verify the role of USP10/YBX1 axis in IS. Additionally, only the effects on neurons were explored, whether the effects on other cells types within the brain such as microglia are worthy of further exploration. Our research provides a theoretical basis for applying natural component TSG in cerebral ischemic injury, suggesting that the therapeutic effect of TSG may benefit from upregulating USP10 and YBX1 levels.

### Ethics Approval and Consent to Participate

The procedures were performed according to the National Institutes of Health Guidelines for Animal Research and approved by the Animal Ethics Committee of Hunan University of Medicine [202211026].

### Availability of Data and Materials

The datasets generated during and/or analyzed during the current study are available from the corresponding author on reasonable request.

### Consent for Publication

N/A.

## Synthesis

Reviewing Editor: Nathalie Ginovart, University of Geneva Decisions are customarily a result of the Reviewing Editor and the peer reviewers coming together and discussing their recommendations until a consensus is reached. When revisions are invited, a fact-based synthesis statement explaining their decision and outlining what is needed to prepare a revision will be listed below. The following reviewer(s) agreed to reveal their identity: NONE.

The reviewer expressed interest in this manuscript as the compound is novel and the results support its effect on reducing injury in this model of ischemic stroke. However, several concerns need to be addressed throughout the manuscript, including clarification of a number of details in the methods, better description of the statistics, number of animals used per group, and power analysis, as well as a discussion of specific concerns raised by the reviewer.

Please find the reviewer's comment below:

Reviewer 1: This is an interesting article that is timely and focused on an important issue. However, the following concerns below should be addressed to improve the potential impact of this study.

1) The title is somewhat misleading with the phrase "improves ischemic stroke injury" and should be edited.

2) Authors are requested to provide information about the number of animals used for the study. How many died in the procedure etc. How many cohorts were used to experiment 1 (Figure 1), what tissues were used for what experiment? A representative timeline image of the design would be helpful,

3) Is the water content measurement method using the % of wet weight, a standard method? If so authors are required to provide the references. Were the full brains or specific regions were used for water content measurement?

4) Authors should provide information about the antibodies used and the microscope used for the immunohistochemistry methods.

5) What was the post hoc analysis performed for the one-way ANOVA used?

6) In methods provide the technique details and/or references for the procedures.

7) Provide the timeline of the experiment. What was the time point for measuring the specific experiment?

8) What was the age of the rats? How many cohorts were used to perform the MCAO study? How were the tissue and homogenates used from the same animal?

9) Please provide the original western blot images with the marking as supplemental figures.

10) In Figure 2f, what is the explanation for the increase in Tom20 in the OGD/R group as compared to the control? That means there is an increase in the mitochondrial content with the injury. How is that possible? What is causing the increased mitochondrial content in the OGD/R group?

11) Figures are not numbered. Its difficult for the reviewer to correlate the text with the figure.

12) The authors need to discuss how TSG could lead to USP10 activation. Is it a direct effect or indirect one since TSG may have multiple effects other than USP10 activation or is it just one of the many common effects due to some master regulatory effect caused by the general effect of TSG?

## Author Response

Dear Editors and Reviewers:

Thank you for giving us the opportunity to revise our manuscript entitled " Tetrahydroxy stilbene glucoside promotes mitophagy and ameliorates neuronal injury after cerebral ischemia reperfusion via promoting USP10 mediated YBX1 stability". We appreciate these helpful suggestions and comments, which have substantially contributed to the improvement of this manuscript. In this revision, we have revised what the reviewers mentioned and highlighted this revision in yellow. We hope that the revised manuscript will be up to your standards and suitable for publication in eNeuro. The main corrections in this paper and the responses to the reviewers' comments are as follows:

Your revision must include the manuscript with new text indicated in a bold or highlighted font to aid the re-review of the manuscript. Separately, please provide a clean copy of the manuscript that includes the title page, which will be published if accepted. Please closely review your manuscript at this time for any final corrections in style or substance. Please consult our Revised Submission Checklist for full details on preparing your revision: https://www.eneuro.org/content/revising-finalizing-manuscript#revisions Response: Thank you very much for your reminding. All changes to this manuscript are highlighted in yellow. As requested, we have provided a clean copy of the manuscript that includes the title page.

In an effort to improve rigor, transparency, and reproducibility, eNeuro strongly encourages the use of estimation statistics. If the use of estimation statistics applies to your research, you may consider including it in your revision. More details and resources can be found in the Information for Authors: https://www.eneuro.org/content/preparing-manuscript#statistics Response: Thank you for your valuable opinions. We tried to use estimation statistics to detect the data in this paper, but this detection method only seems to be suitable for the detection between two groups and is not very suitable for the data in this paper. So I am sorry that we did not modify this part.

Your submission must include publication-quality figures, each in a separate EPS or TIFF (300 dpi) file. Please make sure your figures adhere to style requirements to avoid delays in manuscript processing. Detailed guidelines for figures are available in our Information for Authors: https://www.eneuro.org/content/preparing-manuscript#figures Response: Thank you very much for your reminding. We have read the guidelines for figures carefully and modified the figures in the manuscript as required. See the revised figures for details.

When uploading the revised manuscript, the corresponding author will need to complete the electronic License to Publish form; if not completed during submission, a link to the form will also be available on the author's home page at https://eneuro.msubmit.net.

Response: Thank you very much for your reminding. We have completed and uploaded the electronic License to Publish form.

All authors are invited to submit visually appealing, high-quality images to be used to promote their article, including on the journal homepage. Please submit your images, as well as an image caption and credit information, directly to the peer review system with your revision. Additional details on sizing and image specifications can be found in the Information for Authors: https://www.eneuro.org/content/revising-finalizing-manuscript#image Response: Thank you very much for your invitation. After asking for all authors' opinions, some authors are not very interested in this suggestion, so images for this part are not provided.

Synthesis of Reviews:

Synthesis Statement for Author (Required):

The reviewer expressed interest in this manuscript as the compound is novel and the results support its effect on reducing injury in this model of ischemic stroke. However, several concerns need to be addressed throughout the manuscript, including clarification of a number of details in the methods, better description of the statistics, number of animals used per group, and power analysis, as well as a discussion of specific concerns raised by the reviewer.

Please find the reviewer's comment below:

Reviewer 1: This is an interesting article that is timely and focused on an important issue. However, the following concerns below should be addressed to improve the potential impact of this study.

1) The title is somewhat misleading with the phrase "improves ischemic stroke injury" and should be edited.

Response: Thank you very much for your suggestion. We have changed the title to "Tetrahydroxy stilbene glucoside promotes mitophagy and ameliorates neuronal injury after cerebral ischemia reperfusion via promoting USP10 mediated YBX1 stability" as suggested.

2) Authors are requested to provide information about the number of animals used for the study. How many died in the procedure etc. How many cohorts were used to experiment 1 (Figure 1), what tissues were used for what experiment? A representative timeline image of the design would be helpful, Thank you very much for your advice. A total of 74 animals were used in this study, while 4 of the animals died during experiment, which were excluded. After experiment, rats were euthanized, and brain tissues were immediately collected for subsequent detections. According to experimental requirements, samples were divided into 4 cohorts, which were used for neurological function + TTC measurements, brain water content, pathological examination, and biochemical test, respectively. We have added the information in Part 2 of Materials and methods section as suggested. In addition, according to the suggestion, we have supplemented a representative timeline image of the experimental design in Fig. 1A, pleases see the revised figure for details.

3) Is the water content measurement method using the % of wet weight, a standard method? If so authors are required to provide the references. Were the full brains or specific regions were used for water content measurement? Response: Thank you very much for your advice. The method for detecting brain water content is referred to the previous study [PMID: 3392116], which has been added in Part 6 of Materials and Methods section as suggested. Ischemic hemisphere samples were used for water content measurement. See the revised manuscript for details.

4) Authors should provide information about the antibodies used and the microscope used for the immunohistochemistry methods.

Response: Thank you for your nice opinions. We have added the information of anti-LC3 (PA1-46286, 1:200, Invitrogen) and microscope (BX51, Olympus) in immunohistochemistry in Part 8 of the Materials and Methods section. See the revised manuscript for details.

5) What was the post hoc analysis performed for the one-way ANOVA used? Response: Thank you for your valuable opinions. Tukey's post-hoc test was performed for the one-way ANOVA. We have added the information in the last part of Materials and Methods section.

6) In methods provide the technique details and/or references for the procedures.

Response: Thank you very much for your advice. According to the suggestion, we have supplemented details of the relevant experiments in the Materials and Methods section, and have supplemented several references appropriately. See the revised manuscript for details.

7) Provide the timeline of the experiment. What was the time point for measuring the specific experiment? Response: Thank you very much for your advice. Regarding the timeline of animal experiments, we have previously described in Part 2 of the Materials and Methods section. For a further intuitive reflect, a timeline of the experimental design has been added in Fig. 1A, as aforementioned. See the revised figure for details.

8) What was the age of the rats? How many cohorts were used to perform the MCAO study? How were the tissue and homogenates used from the same animal? Response: Thank you very much for your advice. Rats with 6-8 week-age were used in this study. Animal experiments were divided into 4 cohorts, which were used for neurological function + TTC measurements (n = 5 per group, a total of 25), brain water content (n = 5 per group, a total of 15), pathological examination (n = 5 per group, a total of 25), and biochemical test (n = 5 per group, total 15), respectively. Different cohorts of experiments were performed independently, so tissues for pathological examination and homogenates for biochemical test were derived from different cohorts of animals.

9) Please provide the original western blot images with the marking as supplemental figures.

Response: Thank you very much for your advice. We have provided the original western blot images with the marker as required. See supplementary materials-WB raw data file for details.

10) In Figure 2f, what is the explanation for the increase in Tomm20 in the OGD/R group as compared to the control? That means there is an increase in the mitochondrial content with the injury. How is that possible? What is causing the increased mitochondrial content in the OGD/R group? Response: Thank you very much for your valuable suggestions, which are of great help in improving the preciseness of our manuscript. Studies have demonstrated that mitophagy mediated by PINK1/Parkin signaling is suppressed under cerebral ischemia conditions, while promotion of mitophagy is able to ameliorate cerebral ischemic injury [PMID: 37797433, 32759963]. Therefore, the explanation for the increase of Tomm20 in the OGD/R group may be as follows: When mitophagy occurs, PINK1/Parkin signaling is activated, and PINK1 recruits Parkin to the mitochondria surface to activate Parkin E3 ubiquitin ligase; Parkin can label mitochondrial components such as translocase of the mitochondrial membrane 20 (Tomm20) through ubiquitination, which results in the degradation of these mitochondrial proteins by proteasome; When inhibiting mitophagy, downregulated Parkin lost its function on degradation of Tomm20 via ubiquitination, leading to an increase in the level of Tomm20 [PMID: 24875736]. For example, Lixuan Yang et al. has discovered that mitophagy was repressed in LPS-induced astrocytes, and Parkin in the cytoplasm fails to transfer to the mitochondria, which contributes to the stability of Tomm20; Morinda officinalis lead to ubiquitination and degradation of Tomm20 through promoting the enrichment of Parkin in mitochondria, thereby facilitating mitophagy and mitigating depression-like behaviors in hypertension rats [PMID: 36765376]. In addition, it has been revealed that BDNF induced mitophagy via reducing Tomm20 and increasing LC3II/LC3I, thereby alleviating high-glucose-induced brain microvascular endothelial cell injury [PMID: 30877409].

Similarly, in this study, protein levels of PINK1, Parkin and Tomm20 were detected after mitochondria isolation. Our study discovered reductions of PINK1 and Parkin as well as increase of Tomm20 in mitochondria under OGD/R conditions. However, TSG could promote the transfer of Parkin to mitochondria, which led to the degradation of Tomm20, thereby downregulating its expression. We are very sorry for the confusion caused by lack of clarity, detail and comprehensiveness in our description and picture presentation. We have explained in the Results section that the protein levels of PINK1, Parkin, and Tomm20 were detected after mitochondria isolation. For details, please see the revised Fig. 1H, Fig. 2F, Fig. 5H, and Fig. 6E, and corresponding description of the results. In addition, the above have been explained in Paragraph 3 of the Discussion section. Please see the revised manuscript for details.

11) Figures are not numbered. Its difficult for the reviewer to correlate the text with the figure.

Response: Thank you very much for your suggestions. Figures have been numbered in the upper left corner. See the revised figures for details.

12) The authors need to discuss how TSG could lead to USP10 activation. Is it a direct effect or indirect one since TSG may have multiple effects other than USP10 activation or is it just one of the many common effects due to some master regulatory effect caused by the general effect of TSG? Response: Thank you very much for your valuable advice, which is very helpful to improve the quality of our manuscript. Currently, how TSG leads to USP10 activation is still unclear. It has been found that resveratrol, a TSG analog, disrupted the interaction between Ras-GTPase-activating protein SH3 domain-binding protein 1 (G3BP1) and USP10 through directly targeting G3BP1, thereby enhancing USP10-mediated p53 deubiquitination [PMID: 24998844]. G3BP1 is one of the main components of stress granules (SGs) [PMID: 37580991]. SGs are cytoplasmic aggregates of stalled translational preinitiation complexes induced by various environmental stresses such as heat shock, hypoxia, endoplasmic reticulum stress and oxidative stress [PMID: 31636118]. In ischemia/reperfusion injury, regulating the formation of SGs is a promising intervention for improving self-protection capability of the cells and inhibiting apoptosis [PMID: 32982696]. As a resveratrol analog, whether TSG affects the activation of USP10 through directly interacting with G3BP1 protein or regulates the function of USP10 via combining with other proteins remains to be further explored. We have added the above to Paragraph 4 of the Discussion section as suggested.
